# Acceptable Risk Analysis for Abrupt Environmental Pollution Accidents in Zhangjiakou City, China

**DOI:** 10.3390/ijerph14040443

**Published:** 2017-04-20

**Authors:** Xi Du, Zhijiao Zhang, Lei Dong, Jing Liu, Alistair G. L. Borthwick, Renzhi Liu

**Affiliations:** 1 State Key Laboratory of Water Environment Simulation, School of Environment, Beijing Normal University, Beijing 100875, China; elena_dx@mail.bnu.edu.cn (X.D.); zzhijiao@163.com (Z.Z.); dl@mail.bnu.edu.cn (L.D.); 201521180038@mail.bnu.edu.cn (J.L.); 2 Institute of Environmental & Damages Assessment, Guangdong Provincial Academy of Environmental Science, Guangzhou 510045, China; 3 School of Engineering, The King’s Buildings, The University of Edinburgh, Edinburgh EH9 3JL, UK; Alistair.Borthwick@ed.ac.uk; 4 St Edmund Hall, Queen’s Lane, Oxford OX1 4AR, UK

**Keywords:** risk acceptance, risk perception, psychometric paradigm, environmental risk, questionnaire, tailings ponds

## Abstract

Abrupt environmental pollution accidents cause considerable damage worldwide to the ecological environment, human health, and property. The concept of acceptable risk aims to answer whether or not a given environmental pollution risk exceeds a societally determined criterion. This paper presents a case study on acceptable environmental pollution risk conducted through a questionnaire survey carried out between August and October 2014 in five representative districts and two counties of Zhangjiakou City, Hebei Province, China. Here, environmental risk primarily arises from accidental water pollution, accidental air pollution, and tailings dam failure. Based on 870 valid questionnaires, demographic and regional differences in public attitudes towards abrupt environmental pollution risks were analyzed, and risk acceptance impact factors determined. The results showed females, people between 21–40 years of age, people with higher levels of education, public servants, and people with higher income had lower risk tolerance. People with lower perceived risk, low-level risk knowledge, high-level familiarity and satisfaction with environmental management, and without experience of environmental accidents had higher risk tolerance. Multiple logistic regression analysis indicated that public satisfaction with environmental management was the most significant factor in risk acceptance, followed by perceived risk of abrupt air pollution, occupation, perceived risk of tailings dam failure, and sex. These findings should be helpful to local decision-makers concerned with environmental risk management (e.g., selecting target groups for effective risk communication) in the context of abrupt environmental accidents.

## 1. Introduction

Abrupt environmental accidents occur worldwide; outstanding recent examples include pollution of the Songhua River in China in 2005 after a petrochemical plant explosion, the Deepwater Horizon oil spill in the Gulf of Mexico in 2010, and the Fukushima nuclear accident in 2011. Such incidents can cause considerable damage to the ecological environment, infrastructure, property, human health and wellbeing, institutions (such as schools and hospitals), and economic prosperity, and so are potentially very challenging for government emergency management. With vastly improved communication systems, the growth in public awareness of environmental pollution risk means that an accidental release of pollution can rapidly become a media and political focal point in modern society. Research into the concept of acceptable risk effectively originates from Starr [1] who suggested a way of evaluating societal benefits and technological risks through answering the seemingly simple question: How safe is safe enough? To date, the concept of acceptable risk has been applied in technical safety assessment (e.g., of dams, oil and gas pipelines, chemical industries, etc.) and natural hazard prevention (e.g., earthquakes, floods, landslides, etc.) [2,3,4,5]; however, little research has focused on the acceptable risk of accidental pollution events. 

Approaches to determining acceptable risk include: the use of F-N curves [6], cost-utility analysis [7,8], the life quality index [9,10], and risk matrix [11]. Most of these methods use objective indicators (e.g., mortality, property loss) to analyze technical risk and natural disaster risk, but few consider subjective factors. After Slovic [12] introduced the psychometric paradigm into risk perception research, acceptable risk also became a focus of academic interest in management and psychology. In recent years, well-established psychometric methods have been successfully incorporated into risk assessment [4,13]. Nowadays, the public is viewed as not only the receptor of environmental pollution risk but also the executor of environmental policy and environmental management action. Public understanding of environmental pollution risk and the extent of trust in government control of such risk can affect the behavior of citizens and degree to which state policies are actually implemented. It is therefore necessary for decision makers to understand public attitudes towards accidental environmental pollution risk. 

The study area, Zhangjiakou City, is situated in northwest Hebei Province in China. Zhangjiakou possesses a large number of chemical enterprises and mine tailing ponds; consequently, a total of 4.68 million citizens face the threat of abrupt environmental pollution from three main sources: accidental air pollution, accidental water pollution, and the failure of mine tailings dams. Moreover, Zhangjiakou is located very close to and upwind of Beijing, thus placing a further 21 million people in danger of the sudden release of airborne pollutants. We therefore carried out a questionnaire-based survey of residents of Zhangjiakou, aiming to: (1) evaluate the level of public acceptance of abrupt environmental pollution risks in this region; (2) analyze demographic differences in public attitudes toward abrupt environmental pollution risks; and (3) explore the factors that influence the acceptable risk level tolerated by the public. The findings should be of assistance to environmental regulators required to understand public attitudes towards abrupt environmental pollution risks, and build effective risk communication between the public and the government.

## 2. Materials and Methods

### 2.1. Principles of Acceptable Risk Analysis

The psychometric paradigm is one of the most influential and frequently applied methods in the field of analyzing public risk perceptions. Fischhoff et al. [14] pointed out six potential effect factors related to perceived and acceptable risk: (i) voluntary exposure to a given risk, (ii) familiarity with the risk, (iii) perceived controllability of the risk, (iv) the potential for catastrophic (multiple-fatality) consequences, (v) the immediacy of the consequences, and (vi) the extent of scientific and public knowledge about the consequences. Slovic [12] used a questionnaire to measure the perception of risk and found that characteristic attributes included newness, controllability, severity of consequences, and knowledge about risk. Huang et al. [13] established a risk perception model based on Slovic’s original questionnaire that included independent variables: newness, immediacy of effects, knowledge, benefits, severity of consequences, controllability of risk, and trust in government. It should here be noted that the present research is about risk acceptance of abrupt environmental accidents, and so the benefits and controllability of risk are not involved. The severity of consequences is considered as a risk attribute, and evaluated alongside the possibility of risk. Previous research has shown that demographic differences such as gender, age, marital status, educational level, race, and income may influence public perception of risk or willingness to pay [15,16,17,18,19]; demographic variables are also considered in the present survey questionnaire.

### 2.2. Questionnaire

The questionnaire created to measure public attitudes toward accidental environmental pollution risk (Table A1) was divided into three parts; namely, demographic variables, perceived risks, risk attitude factors, and risk acceptance. Demographic variables included sex, age, education, occupation, monthly income, and proximity to risk sources. In terms of perceived risk, three categories of abrupt environmental pollution risks were considered: accidental water pollution, accidental air pollution, and tailings dam failure. Twelve items were used to measure public perception of these risks in terms of both the possibility and the consequences of abrupt environmental accidents, and each item was evaluated on a five-point Likert scale (1, strongly disagree; 2, disagree; 3, neutral; 4, agree; 5, strongly agree). The score assigned to each item was obtained as the product of its possibility and consequence. Each perceived risk was evaluated by the mean score of items involving accidental water pollution (Q1–Q5), accidental air pollution (Q6–Q8), and tailings dam failure (Q9–Q12), respectively. Risk attitude factors included: public knowledge of environmental pollution risks (Q13); familiarity with environmental measures carried out by government agencies (Q14); satisfaction with environmental management (Q15) (evaluated using a five-point Likert scale); and experience of abrupt environmental accidents (Q16), which was dichotomous variable. To gain a further understanding of people’s attitudes toward environmental risk, questions related to concern about abrupt environmental accidents and willingness to pay for measures to reduce risk were also included in the questionnaire. Explanations of particular terminology and relevant cases of accidental environmental pollution were included with the questionnaire to provide background reference material to help the survey respondents when making self-assessments and answering the questions. 

### 2.3. Analytical Approaches

The data analysis procedure comprised four steps. Firstly, we examined public attitudes toward accidental environmental pollution risks in different districts. Secondly, we analyzed the influence of demographic characteristics on public acceptance of accidental environmental pollution risks; the Chi-square test was used to analyze the variance of risk acceptance in each categorized group of respondents. In the third step, the influence of perceived risk and risk attitude factors (i.e., risk knowledge, familiarity with environmental management, satisfaction with environmental management, and risk experience) on risk acceptance was analyzed. Since the data on perceived risks did not fit a normal distribution, the Mann-Whitney U test was used to test the variance of perceived risk [20]. The influences of risk attitude factors on risk acceptance were examined using the Chi-square test. Through the second and third step, the significant factors were identified. In the last part, the multiple regression analysis was used to explore the correlation between demographic characteristics, risk attitude factors, and public risk acceptance. The dependent variable in the regression analysis was “risk acceptance”, as a yes/no response (Q19). Due to the binary nature of the predicted variable in this study, logistic regression was utilized which is suited to dichotomous variables. Logistic regression can therefore be applied to variables that are discontinuous or qualitatively derived, and does not require assumptions of normality and homogeneity of variance [21]. The general equation is as follows:

(1) $$\mathrm{Logit}\left(p\right)=\mathrm{Ln}\left(\frac{p}{1-p}\right)=b_{0}+b_{1}x_{1}+\cdots +b_{k}x_{k}$$

where p represents the odds of falling into the “1” category, x_1_, x_2_, …, x_k_ is the series of independent variables, b_1_, b_2_, …, b_k_ are coefficients, and b_0_ is the intercept. 

Certain group sizes were inadequate for statistical analysis, so we categorized respondents according to sample size and characteristics. Age was divided into four groups: ≤20 y, 21–40 y, 41–60 y, >60 y, noting that the sixth population census in 2010 found the average age of residents in Zhangjiakou was 38 and predicted to increase. Education was divided into four categories: primary school and below, junior high school, high school, and college or above. Occupation was separated into two groups: people working in the public sector (e.g., administration, public institutions, and social organizations) and people in other occupations (i.e., employees of private enterprises, students, farmers, self-employed, and others). The average per capita disposable income in Zhangjiakou in 2014 was about 1117 yuan per month, and so we classified monthly income into two levels: ≤1000 RMB and >1000 RMB. Respondents were also categorised by proximity to risk sources into four groups: ≤500 m, 500–3000 m, 3000–5000 m, >5000 m. 

The reliability and internal consistency of the perceived risks scale were tested using Cronbach’s α coefficients. Data analysis was performed using the statistical package SPSS 20.0 (Armonk, NY, USA).

### 2.4. Study Area and Samples

**Figure 1:**
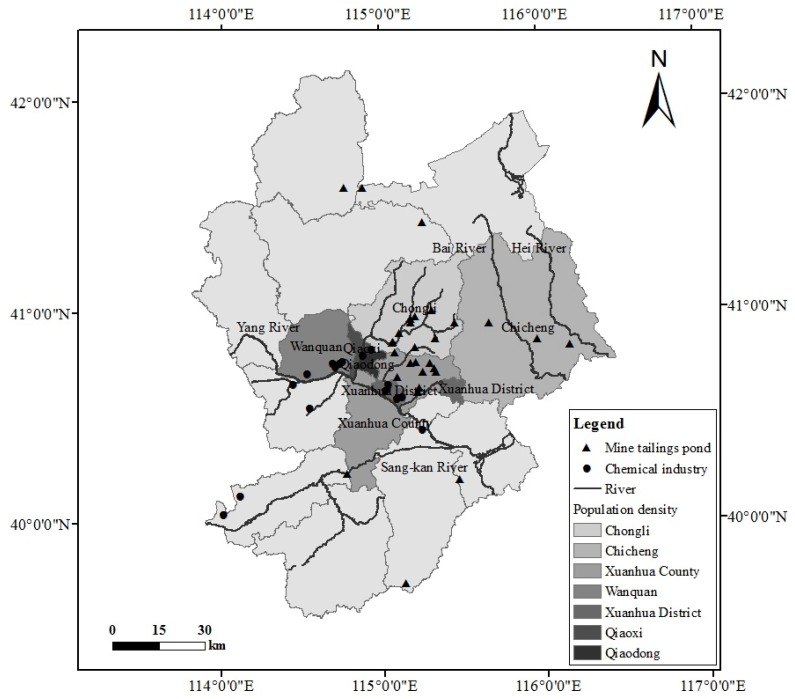
Study area and main accidental pollution risk sources within Zhangjiakou City, China.

Zhangjiakou is a relatively sparsely populated, less developed city, situated in northwest Hebei Province and upwind of Beijing, China. According to the Statistical Yearbook [22], at the end of 2014, Zhangjiakou had a total population of 4.68 million and overall gross domestic product (GDP) of 135.85 billion RMB. Sixteen chemical enterprises that have the potential to cause air pollution accidents are located in the vicinity of Zhangjiakou; five are located in Wanquan, four in Xuanhua District, two in each of Qiaodong, Huai’an, and Yangyuan, and one in Zhuolu (see Figure 1). Also, as a mountainous region, Zhangjiakou possesses abundant metal mineral resources (e.g., iron, gold, silver, and lead zinc) and so faces a considerable acute pollution threat from mine tailings ponds. Of these, about 250 iron mine tailings ponds and 29 heavy metal mine tailings ponds are located within the selected area [23], most of them along the Yang River and its biggest branch, the Qingshui River (see Figure 1). 

In previous studies, we have analyzed the risk for tailings pond pollution accidents in Zhangjiakou [23]. In order to consider the integrated effect of 16 chemical enterprises and 29 heavy metal mine tailings ponds, we selected the following five districts and two counties of Zhangjiakou exposed to pollution hazards as comprising the region in which to conduct the present survey: Qiaodong District, Qiaoxi District, Xuanhua District, Xuanhua County, Wanquan District, Chongli District, and Chicheng County. Of these, Qiaodong and Qiaoxi are situated in the central city area and have higher population density than the other areas considered. Chemical industries are relatively dense in Wanquan and Xuanhua, and so their residents may be influenced by potential environmental risks. Chongli and Chicheng are situated in mountainous area, with Chongli possessing many mine tailings ponds.

The survey was conducted in October, 2014 and involved 900 randomly sampled residents. For each selected district/county, 5% of residential communities/villages were randomly selected by their names from the Statistical Yearbook [21], and 10% households within the selected communities/villages were randomly chosen by the registered head of a household from the local census lists. To help eliminate misunderstandings, the survey questionnaires were distributed in person so that clarification could be given to the participants if need be, and the respondents could communicate with us directly about local environmental problems. This helped ensure a high response rate, with 870 valid questionnaires returned, including 115 from respondents in Qiaodong, 185 from Qiaoxi, 172 from Xuanhua District, 134 from Xuanhua County, 104 from Wanquan, 68 from Chongli, and 92 from Chicheng (see Figure 1). 

## 3. Results

### 3.1. Sample Characteristics

The results showed 40.3% of the 870 valid survey respondents were male. Most respondents (70.0%) were between 20 and 50 years of age. The educational level in this region was relatively underdeveloped with 42.9% of the participants having only had a junior high school education or below, and 30.2% at least a college education. 28.5% of the survey respondents were self-employed, 21.6% were farmers, and 17.4% worked in administrative departments and public institutions. 89.0% of respondents had a monthly income below 3000 RMB. 9.0% of respondents lived within 500 m of a chemical plant or tailing pond, whereas 53.4% lived more than 5000 m away. Cronbach’s α coefficient relating to the scale for perceived risk is 0.967, which confirms that the questionnaire was reliable and internally consistent. About 57.1% of respondents in this survey stated that they were prepared to accept the present level of accidental environmental risk. The average perceived risks by respondents was 14.12 for accidental water pollution, 14.28 for accidental air pollutions, and 13.50 for tailings dam failures. According to the survey, when environmental accidents occurred, the greatest public concern was impact on health, followed by damage to the environment and property loss.

**Figure 2:**
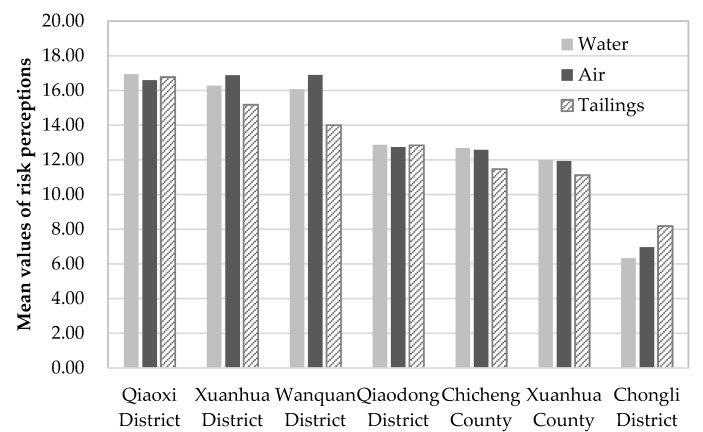
Histogram of mean values of risk perceptions for seven districts and counties in Zhangjiakou City, China.

The results indicated that residents of Qiaoxi, Xuanhua District and Wanquan perceived higher risks, whereas those in Chongli had the lowest risk perceptions (see Figure 2). Public perceptions of the three types of abrupt environmental risks were all high level in Qiaoxi, with the highest perceptions of accidental water pollution risk (M_water_ = 16.93) and tailings dam failures (M_tailings_ = 16.77) in the seven districts surveyed. Residents of Wanquan demonstrated the highest perception of accidental air pollution risk (M_air_ = 16.89) and high perception of accidental water pollution risk (M_water_ = 16.08). Results from Xuanhua District and Qiaoxi were the same, with respondents having higher perceived risk of abrupt water pollution (M_water_ = 16.27) and abrupt air pollution (M_air_ = 16.88). 

### 3.2. Differences in Public Acceptance among Demographic Groups

As shown in Table 1, the age, occupation, income, sex, and education of people influence their risk acceptance level. Male respondents indicated much higher risk acceptance than females. Only 53.9% of female respondents considered the abrupt environmental risk in their neighborhood to be acceptable, while 61.8% of men tolerated the risk. Older people were more willing to accept the risk. People over 60 years old showed the highest risk acceptance ratio (75.0%). People with lower education level had higher risk acceptance, while people who went to a college were more reluctant to accept the risk. Compared to other occupations, people who worked in public service tended to consider the risk unacceptable, with only 45.0% of people willing to tolerate the risk. People who had income lower than average showed higher risk acceptance than those with higher income. The effect of distance from the risk source in this case was insignificant. 

### 3.3. Influence of Perceived Risk and Risk Attitude Factors

The perceived risk of environmental pollution accidents, public satisfaction with environmental management, and risk experience can influence public risk acceptance significantly. From Table 2, it can be seen that people who perceived lower risk were more willing to accept the risk. The mean value of perceived abrupt water risk of people who considered the risk unacceptable was much higher than that of people who accepted the risk (Z = 8.726, *p* ≤ 0.001). The same conclusion can be drawn with respect to the perceived risk of abrupt air pollution and tailings dam failure. 

The knowledge of environmental risk and environmental management were relatively low, with only 3.7% of respondents indicated they knew very much about environmental risk (choosing “strongly agree” on Q13), while 16.8% had no risk knowledge at all (choosing “strongly disagree”). Only 2.5% of respondents were very familiar with the environmental measures carried out by government (choosing “strongly agree” on Q14), while 9.0% knew nothing about environmental measures. 2.5% of people felt satisfied with the environmental management (choosing “strongly agree” on Q15), while 6.3% stated strong dissatisfaction. 37.7% of people had experienced abrupt environmental events in this region. 

To investigate the effect of risk attitude factors on risk acceptance, the responses on risk knowledge, satisfaction, and familiarity with environmental management were divided into three levels: low (“strongly disagree” and “disagree”), medium (“neutral”), high (“strongly agree” and “agree”). As shown in Table 3, public satisfaction with environmental management and risk experience had a significant influence on risk acceptance. The more people felt satisfied with the environmental measures carried out by the government, the more they were willing to accept the risk. 80.2% of respondents with high level of satisfaction indicated that the environmental risk acceptable, whereas the ratio was only 56.4% and 38.2% for people with medium level and low levels of satisfaction (χ^2^ = 83.784, *p* ≤ 0.001). People who had experienced environmental pollution accidents exhibited lower risk acceptance than others (χ^2^ = 23.610, *p* ≤ 0.001). 

However, the effects of risk knowledge and public familiarity with environmental management on risk acceptance were not significant. From the investigation, we found many people would rather choose medium than making the choice between agreeing and disagreeing. The values listed in Table 3 indicate that people with low-level knowledge of risk had a higher acceptable risk ratio than people with high-level knowledge of risk. People with high-level familiarity with environmental management were more willing to accept the risk than those with low-level familiarity.

### 3.4. Regression Analysis of Risk Acceptance

By means of the chi-square test and the independent sample *t*-test, significant factors affecting risk acceptance were identified, including sex, age, education, occupation, income, perceived risk of abrupt water pollution, perceived risk of abrupt air pollution, perceived risk of tailings dam failure, satisfaction with environmental management, and risk experience. As a prerequisite to the logistic regression analysis, the categorical variables were reclassified as introduced in Section 2.3. Table 4 lists definitions and values assigned to the variables as well as results from the logistic regression analysis of public acceptance associated with abrupt environmental risks in the study region. The results showed that, of the 10 selected factors, 5 important factors influenced public risk acceptance: sex, occupation, satisfaction with environmental management, perceived risk of abrupt air pollution, and perceived risk of tailings dam failure. Males were found to be more willing to accept environmental risk than females (*p* < 0.05). Compared to people with other occupations (i.e., employees of private enterprises, students, farmers, self-employed, and others), those people who worked in public service (i.e., administration, public institutions, and social organizations) tended to consider the environmental risk unacceptable (*p* < 0.05). People who were not satisfied with the environmental management or were neutral were more reluctant to accept risk than those people who were satisfied with the environmental management (*p* < 0.001). Perceived risks of abrupt air pollution and tailings dam failure were negatively correlated with public risk acceptance (*p* < 0.05), implying that people who perceived higher risks of these two environmental accidents had lower risk acceptance. 

## 4. Discussion

The districts selected for this survey are vulnerable to abrupt environmental risks from nearby chemical industries and tailings ponds. Residents in Wanquan and Xuanhua District (where there is dense industry) perceived higher risks than residents in other areas, especially with regard to the risk of accidental water pollution and accidental air pollution. People living in the mountainous areas of Chongli and Chicheng were highly influenced by tailings dam failures. Being located farther away from risk sources, the residents of Xuanhua County exhibited lower risk perception than those who lived in Xuanhua District. Respondents from nearby central districts of Qiaodong and Qiaoxi reported equivalent levels of risk knowledge and public trust levels, but residents of Qiaoxi demonstrated much higher perceptions of environmental risks than those of Qiaodong. Although there were relatively few risk sources in both districts, people in Qiaoxi indicated that they were influenced by the chemical industries and tailings ponds because of close proximity to Wanquan and Chongli. The survey revealed that most of the respondents in Chongli were farmers and had less knowledge of environmental risks, which may be the reason for the extremely low risk perception in that area.

The risk level acceptable to the public may be influenced by two types of factor. The first type is inherent to different groups of people and relatively unchanging, and relates to human attributes, personalities, and social status. For instance, people of different sex, age, educational level, occupation, and income may have different perception and sensibility to risk. Many studies have observed sex differences in risk perception, finding that women exhibit more concern about technological risks than men [18,24,25]. The results from the present study concur with this finding. Previous research has shown that women perceive greater probabilities of negative consequences from engaging in risky behavior, whereas men obtained more enjoyment from risk while assuming that potential negative outcomes would not occur [26]. Thus, it appears likely that women perceive higher possibility of a given risk and greater severity of its consequence. The effect of age on risk acceptance has proved controversial in other studies [15,27]. In the present study, people over 60 years and people under 20 years of age were more willing to accept the environmental risk. These two groups of people had lower knowledge about environmental risk and were not very familiar with the environmental management systems in place. It has previously been established that environmental concern is positively associated with social class as indicated by education, income, and occupational prestige [28]. Our findings strongly support this social class hypothesis. People who had higher education, namely college and above, showed lower risk acceptance than others. The difference of risk acceptance was also reflected in people in different income brackets. People with lower incomes than average exhibited a significantly higher ratio of risk acceptance. Huang et al. [13,29] found that people with lower income formed the more sensitive group to nuclear accidents. However, in our case study, environmental risk was not their first concern. Most people with lower incomes showed less interest in knowing about environmental risk and environmental measures carried out by local government. With jobs, family, and other demands of daily living, their lives are filled with more immediate concerns than environmental risk [30]. Further studies are needed to examine whether risk acceptance is related to the income. Besides, we found that people who work in public service institutions, turn out to be more sensitive to abrupt environmental pollution accidents, unlike Huang et al.’s study [29]. In our case, the samples of people who work in public service institutions were mostly from the local environmental protection bureaus. These people are considered to have more knowledge about environmental risks and have more direct connection with environmental risks; therefore, they may be more sensitive to environmental accidents. We considered this phenomenon as a beneficial for improved environmental management.

The second type of factors arise from external circumstances, including objective conditions (e.g., geographical position, risk attribute) and communication of environmental information (e.g., experience of environmental accidents, risk knowledge, risk perception). These impact factors are cumulative and can change with time. In this study, public perception of risks, experience of risks, and satisfaction with environmental management turned out to be significantly associated with risk acceptance. When people felt an increased threat of risk, they would begin to consider the risk around them to be unacceptable. Böhm and Pfister [31] found that perceived risk depend on the type of potential consequences. Katsuya proved that lower levels of perceived accident likelihood led to higher acceptability of nuclear power [32]. The uncontrollability and severity of abrupt environmental accidents increase the fear experienced by people, and serve to heighten defense mechanisms. In the present investigation, people were mostly concerned with the impact of an environmental accident on human life and health. Many empirical studies have shown a strong correlation between public attitudes and perceived risk. Renn [33], Shimooka [34], and Huang et al. [29] found that after experiencing the nuclear power plant accident, the number of opponents significantly increased. The risk experience can not only influence public attitudes or risk acceptance but also other factors. Public perception of environmental risk and knowledge about the risk increased significantly, while public trust in government declined after the environmental accidents [29]. Experiences that threaten people’s life or health may make potential victims more sensitive to environmental risk in the future, thereby consciously or unconsciously absorbing relatively more information during their daily lives. Weyman and Kelly [35] found that members of the public tend to overestimate the risks they are familiar with, rather than unfamiliar risks. This familiarity could come from the experience of similar risks or knowledge gleaned from media and other information sources. In this study, we found that public knowledge about risk and familiarity with environmental management were not strongly related to risk acceptance, which concurred with other findings [32,36,37]. Other researchers also found people having trust in authorities and the management responsible for the technology or the plant perceived fewer risks than people not having trust [13,38,39,40]. We surveyed public familiarity and satisfaction with environmental management in this study, and the satisfaction with environmental management was negatively correlated with public risk acceptance. We believed if the public were very familiar and satisfied with environmental management they would have more faith in government to control the environmental accidents effectively.

Through the logistic regression analysis, five significant factors were retained in the final model: sex, occupation, satisfaction with environmental management, perceived risk of abrupt air pollution, and perceived risk of tailings dam failure. These were considered the most important factors that influenced public acceptance in this case. Members of the public hold different perceptions of different kinds of risks [41]. In the present research, we found that the perceived risk of accidental water pollution had less effect on public risk acceptance than accidental air pollution or tailings dam failure in the Zhangjiakou area. The public may perceive a higher level of threat from risks that directly influence their lives.

In environmental management, the second type of impact factors may be more easily controlled and applied than the first type. With this in mind, regulators should strengthen information disclosure policy and improve education on environmental risks so that the public can obtain more information about environmental risks. Furthermore, risk communication plays an important role in environmental management. The government is obliged to guide the public to develop an informed understanding of abrupt environmental risk and thus be able to take effective action to prevent and decrease damage from an accidental environmental event. After such an event, the government should act to calm victims and take measures to diminish future worries and concerns. In our study, public satisfaction with environmental management is the most significant factor influencing risk acceptance. Trust and credibility are based on three determinants: knowledge and expertise, openness and honesty, and concern and care [42]. Hence, public trust can be gradually established by means of improved risk communication. 

The questionnaire survey has proven to be a direct and effective way to test public attitudes and opinions. One limitation is that respondents’ understanding of the questionnaire can influence their score. The value of risk knowledge is mostly based on people’s self-assessment. To make sure the questionnaire is understandable for people with different educational levels, a pre-investigation is necessary. Furthermore, the logistic regression model may not suitable for other regions. Further research is necessary to explore the impact factors associated with risk acceptance.

## 5. Conclusions

The purpose of this study is to characterize the acceptable risk level to local residents of abrupt environmental risk in selected districts of Zhangjiakou City, Hebei Province, China. Demographic and regional differences have been examined regarding risk attitude and impact factors associated with risk acceptance. Useful information is provided for risk assessment on a regional scale, and suggestions made for decision makers and regulators to build a better risk communication with public.

Regional differences are very significant in the Zhangjiakou City area. Residents of Qiaoxi have the highest perception of abrupt water pollution risk and abrupt tailings dam failure, those in Wanquan and Xuanhua District have the highest perception of abrupt air pollution risk, whereas residents in Chongli District have the lowest perception of all three abrupt environmental risks considered. People with different sex, age, education, occupation, and income showed different risk acceptance. Females, people aged between 21–40 years, people with higher levels of education, public servants, and people with higher income had lower risk tolerance. People who perceived higher risk found the environmental risk around them unacceptable. People with low-level risk knowledge, high-level familiarity and satisfaction with environmental management, and people who had no experience of environmental accidents showed higher risk tolerance. The logistic regression analysis showed that public satisfaction with environmental management was the most significant factor associated with risk acceptance, followed by perceived risk of abrupt air pollution, occupation, perceived risk of tailings dam failure, and sex.

## Figures and Tables

**Table 1:** Differences in risk acceptance among demographic groups.

Variable		N (%)	Acceptable (%)	χ^2^
Sex	Male	351 (40.3%)	61.8	5.300 *
	Female	519 (59.7%)	53.9	
Age	≤20	65 (7.5%)	63.1	15.673 **
	21–40	431 (49.5%)	53.4	
	41–60	282 (32.4%)	55.7	
	>60	92 (10.6%)	75.0	
Education	Primary school and below	95 (10.9%)	66.3	7.936 *
	Junior high school	278 (32.0%)	57.9	
	High school	234 (26.9%)	59.4	
	College or above	263 (30.2%)	51.0	
Occupation	Public service	169 (19.4%)	45.0	12.654 ***
	Others	701 (80.6%)	60.1	
Income	≤1000	382 (43.9%)	63.4	10.773 **
	>1000	488 (56.1%)	52.3	
Distance	<500	78 (9.0%)	47.4	6.650
	500–3000	208 (23.9%)	63.5	
	3000–5000	119 (13.7%)	55.5	
	>5000	465 (53.4%)	56.3	

Significance: * *p* ≤ 0.05; ** *p* ≤ 0.01; *** *p* ≤ 0.001.

**Table 2:** Effect of perceived risk on risk acceptance.

Variable	Unacceptable		Acceptable		Z
	Mean	SE	Mean	SE	
Perceived risk (water)	16.80	0.41	12.10	0.35	−8.460 ***
Perceived risk (air)	17.44	0.48	11.90	0.38	−9.115 ***
Perceived risk (tailings)	16.51	0.41	11.24	0.37	−8.998 ***

SE: Standard error of mean; Z: Statistics of Mann-Whitney U test; Significance: *** *p* ≤ 0.001.

**Table 3:** Effect of risk attitude factors on risk acceptance.

Variable		N (%)	Acceptable (%)	χ2
Knowledge	Low	380 (43.7%)	59.2	1.224
	Medium	333 (38.3%)	55.3	
	High	157 (18.0%)	56.1	
Familiarity	Low	435 (50.0%)	57.5	1.705
	Medium	322 (37.0%)	55.0	
	High	113 (13.0%)	61.9	
Satisfaction	Low	249 (28.6%)	38.2	83.784 ***
	Medium	404 (46.4%)	56.4	
	High	217 (24.9%)	80.2	
Experience	No	542 (62.3%)	63.5	23.610 ***
	Yes	328 (37.7%)	46.6	

Significance: *** *p* ≤ 0.001.

**Table 4:** Logistic regression analysis and definitions of categorical variables.

Variable	B	S.E.	Wals	*p*	OR	Variable Definition
Satisfaction 1	−1.716	0.224	58.450 ***	0.000	0.180	“1” = not satisfied with environmental management; “0” = otherwise
Satisfaction 2	−0.968	0.207	21.946 ***	0.000	0.380	“1” = neutral; “0” = otherwise
Perceived Risk (air)	−0.041	0.014	8.170 **	0.004	0.960	Factor perceived risk of abrupt air pollution
Occupation	−0.434	0.190	5.192 *	0.023	0.648	“1” = working in public service; “0” = otherwise
Perceived Risk (tailings)	−0.030	0.015	4.159 *	0.041	0.970	Factor perceived risk of tailings dam failure
Sex	0.316	0.156	4.085 *	0.043	1.371	“1” = male; “0” = female
Intercept	2.254	0.231	95.066 ***	0.000	9.528	/

B: Coefficient; S.E.: Standard error; Wals: Wald statistics; OR: Odds ratio. Significance: * *p* ≤ 0.05; ** *p* ≤ 0.01; *** *p* ≤ 0.001.

## Appendix A

**Table A1:** Survey questionnaire.

Question ID	Description of Questions
Perceived risks	To what degree do you believe the possibility of the following events happening and the severity of the consequence?
Water pollution	If hazardous substances entered rivers, lakes and underground water suddenly,
Q1	It would make you feel uncomfortable and affect your normal life (e.g., diarrhea, dermatosis, infections)
Q2	It would make tap water or well water discolored and malodorous.
Q3	It would make river water and lakes discolored and malodorous, and kill a large amount of fish.
Q4	It would lead to the death of rare animals and plants in nature reserves and pollute springs and streams.
Q5	It would lead to withering of crops and fruit trees, and decreased production and increased death rates of farmed fish and shrimp.
Air pollution	If hazardous substances entered the atmospheric environment suddenly,
Q6	It would make you feel uncomfortable and affect your normal life (e.g. making you dizzy, vomit, or feel poisoned).
Q7	It would lead to the deterioration of air quality, decrease of birdlife and death of precious plants in nature reserve.
Q8	It would lead to withering of crops and fruit trees, and decreased production and increased death rates of domestic animals.
Tailings dam failure	If tailings dam broke and leaked out hazardous substances suddenly,
Q9	It would damage your health and affect your normal life.
Q10	It would block rivers and lakes, lead to the death of fish and shrimp, and make the river water polluted and malodorous.
Q11	It would pollute nature reserves and tourist resorts.
Q12	It would inundate or damage villages, factories, farmland, orchards and farms.
Risk attitude factors	
Q13	To what extend do you know about environmental risk?
Q14	To what degree are you familiar with the prevention measures of environmental risk that the government has carried out?
Q15	To what degree are you satisfied with the government in its efforts to manage environmental risk?
Q16	Have you ever experienced any of the environmental events mentioned above?
Q17	How much money are you willing to pay to avoid any of these environmental accidents? (yuan)
Q18	What do you worry about when an environmental accident happens?
Q19	Do you consider the environmental risk level around where you live to be acceptable or not?
